# Orthogonalization of Regressors in fMRI Models

**DOI:** 10.1371/journal.pone.0126255

**Published:** 2015-04-28

**Authors:** Jeanette A. Mumford, Jean-Baptiste Poline, Russell A. Poldrack

**Affiliations:** 1 Center for Investigating Healthy Minds at the Waisman Center, University of Wisconsin—Madison, Madison, WI, USA; 2 Helen Wills Neuroscience Institute, Brain Imaging Center, University of California, Berkeley, CA, USA; 3 Department of Psychology, Stanford University, Palo Alto, CA, USA; Centre de Neuroscience Cognitive, FRANCE

## Abstract

The occurrence of collinearity in fMRI-based GLMs (general linear models) may reduce power or produce unreliable parameter estimates. It is commonly believed that orthogonalizing collinear regressors in the model will solve this problem, and some software packages apply automatic orthogonalization. However, the effects of orthogonalization on the interpretation of the resulting parameter estimates is widely unappreciated or misunderstood. Here we discuss the nature and causes of collinearity in fMRI models, with a focus on the appropriate uses of orthogonalization. Special attention is given to how the two popular fMRI data analysis software packages, SPM and FSL, handle orthogonalization, and pitfalls that may be encountered in their usage. Strategies are discussed for reducing collinearity in fMRI designs and addressing their effects when they occur.

## Introduction

When regressors in a general linear model (GLM) are orthogonal to each other, they do not share any descriptive variability and are completely uncorrelated. In contrast, collinearity occurs when there is pairwise correlation between either single regressors or linear combinations of multiple regressors. Eq 1 shows two examples of perfect collinearity.

$$\begin{array}{cc} {}[\begin{array}{ccc} 1 & 2 & 0\\ 1 & 2 & 0\\ 0 & 0 & 1\\ 0 & 0 & 1 \end{array}]: & c_{2}=2c_{1}\\ {}[\begin{array}{ccc} 1 & 0 & 2\\ 1 & 0 & 2\\ 0 & 1 & 4\\ 0 & 1 & 4 \end{array}]: & c_{3}=2c_{1}+4c_{2} \end{array}$$ (1)

**Fig 1:**
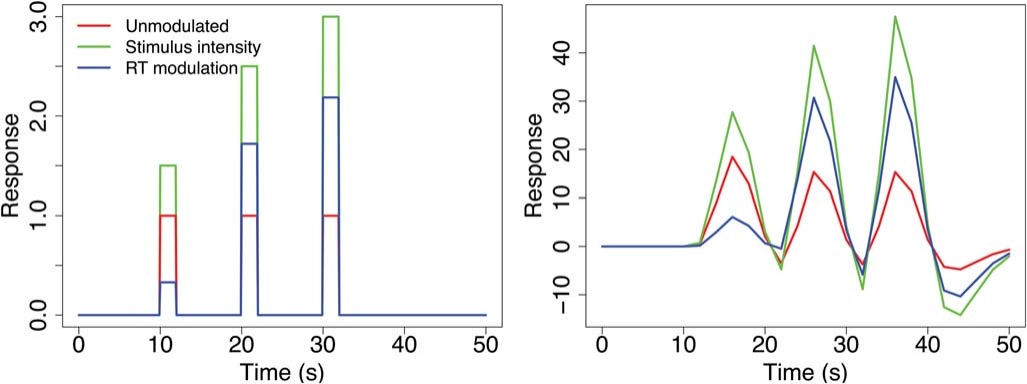
How parametrically modulated regressors are constructed. The left graph shows the unconvolved regressors for 3 trials, where each trial for stimulus intensity and RT is modulated by the appropriate value. The right graph shows the convolved regressors and all pairwise correlations of regressors are above 0.80, indicating possible collinearity.

On the top, the first regressor is perfectly correlated with the second and on the bottom the third regressor is perfectly correlated with a linear combination of the first two. Near-collinearity occurs when the correlation between linear combinations of regressors is high but not perfect. For example, in an fMRI experiment where a stimulus is always followed 2s later by feedback, the stimulus- and feedback-specific regressors will be highly correlated due to the blurring effect of the hemodynamic response. This can also occur when a set of trials has two sets of parametrically modulated values to be modeled. For example, when the model includes a regressor modeling response times (RT) along with a parametric regressor that has a direct effect on RT, such as stimulus intensity; because the onset times are identical for both regressors, the resulting parametric regressors will be correlated. Fig 1 shows how the parametrically modulated regressors are created and illustrates that when RT and the task parameter are highly correlated, the resulting parametrically modulated regressors will also be correlated, in this case the correlation is greater than 0.80. Correlation between regressors is also common in second level studies; for example, age and task performance regressors are likely to be correlated. It should also be noted that collinearity can impact contrasts of parameter estimates as well as individual model parameters [1]. One way to think of this is to re-parametrize the model such that the contrast of effects to be tested is coded with one regressor, and to consider how much correlation there is in the rest of the model with this regressor. Notably, the overall model fit is not impacted by the collinearity in the model, but the quality of the parameter estimates will suffer, in terms of variability.

**Fig 2:**
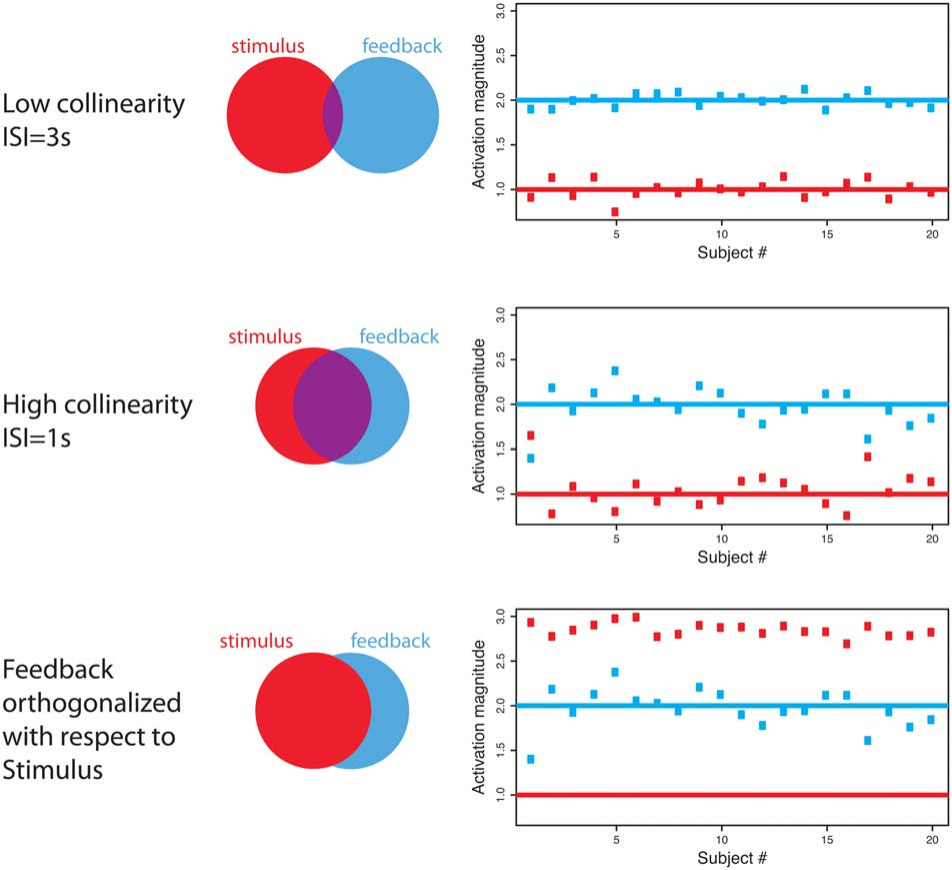
Illustration of parameter estimate behavior for 20 simulated subjects when model has low collinearity (top), high collinearity (middle) and high collinearity where the feedback regressor is orthogonalized with respect to the stimulus regressor (bottom). The Venn diagrams indicate how the variability is distributed across the 2 regressors where red is unique to stimulus, blue is unique to feedback and purple is shared. The rightmost column shows the parameter estimates for stimulus (red) and feedback (blue) across 20 subjects, where the solid lines indicate the true values. With low collinearity the variability of the parameter estimates is much smaller than the high collinearity case, although in both cases the estimates are unbiased on average. Orthogonalization does not change the feedback parameter estimates (middle and bottom blue data points are identical), but the stimulus feedback has lower variability and the estimate is much higher.

Models with perfect collinearity are not estimable because there is an infinite number of solutions that could fit the data equally well. This is comparable to solving the problem of identifying 2 numbers that sum to 10 (5 and 5, -50 and 60, etc.). With near-collinearity, the model is estimable but the resulting parameter estimates are highly variable. Fig 2 shows three different modeling examples for the experiment with a 2s long stimulus followed by feedback for low collinearity (top), high collinearity (middle) and high collinearity with an orthogonalized regressor (bottom). Notice that often we are not concerned by identification of all parameters of the model, but rather with some (linear) combination of these. For now we will focus on individual parameters for simplicity. The orthogonalized case will be discussed shortly, but to understand the impact of collinearity, the current focus is on the top two rows of the figure. The Venn diagrams illustrate the descriptive variability of each regressor, or explanatory variable, in terms of which variance is unique (red and blue) versus shared between the two variables (purple). The rightmost plots show activation estimates for 20 simulated subjects for stimulus and feedback, where the solid lines indicate the true activation strengths. In the low collinearity case (top row, Fig 2), each covariate has a large portion of unique descriptive variability, which leads to activation estimates with a low variance across subjects. In the high collinearity case, with an ISI of 1s (middle, Fig 2), when one parameter estimate within subject is unusually high this is offset by an unusually low estimate for the other parameter, in order to maintain a good overall model fit. For example, the first subject’s stimulus estimate is much higher than the true mean and the feedback estimate is much lower. What is crucial to recognize (and often misunderstood) is that general linear model parameter estimates are unbiased in the case of near-collinearity, but have inflated variance; the standard method of GLM parameter estimation automatically removes the effects of shared variability, so that each effect is adjusted for all others. In the example above, this would allow the stimulus parameter to be interpreted as unique stimulus activation adjusted for feedback activation and likewise the estimated feedback activation is adjusted for the stimulus-related effect. If one wishes to look at the common variance explained by either regressors, an F statistic should be employed.

A common misconception is that orthogonalization is necessary and/or optimal to address the effects of collinearity. Whereas the standard GLM removes the shared variability from all of the individual regressors, orthogonalization instead assigns the shared variability to one of the regressors and is accomplished by regressing one covariate on the other and extracting the residual. In the example, in order to orthogonalize feedback with respect to stimulus, the feedback regressor would be replaced with the residual from the following GLM: *feedback* = β_*_ *stimulus*+*ϵ*. The bottom row of Fig 2 illustrates the orthogonalized model and, importantly, the interpretation of the stimulus effect in this model is “stimulus *unadjusted* for feedback”. In other words, the parameter estimate for stimulus is the same as what would be obtained if feedback was not even in the model (though the residual variance may differ between the model with orthogonalized feedback and feedback omitted). Since collinearity is high, the unadjusted stimulus effect is roughly the sum of the stimulus and feedback effect sizes (1+2 = 3). In addition, since more descriptive variability is assigned to the stimulus regressor, this regressor now explains both what is common to feedback and stimuli as well as what is specific to stimulus. In addition, the variance of the estimate is much smaller in this model, i.e., the subject-specific estimates are less variable across subjects. The overall goodness of fits for the models with and without orthogonalization are identical, as are the parameter estimates for the feedback covariate (blue points, middle and bottom rows, Fig 2). This occurs since the same descriptive variability is available for both models, in other words, the portion in the Venn diagrams unique to feedback (blue) is the same in both models. A related example written in Python illustrating orthogonalization using can be found in http://nbviewer.ipython.org/github/jmumford/orthogonalizaton_ipynb/blob/master/orthogonalization.ipynb.

Overall, the only effect of orthogonalization in the example is to change the interpretation of the parameter associated with the stimulus. This effect of orthogonalization is often overlooked and/or misunderstood when interpreting the parameters of the model, which produces a misleading result. The test statistic for a parameter estimate consists of the estimated mean divided by the estimated variance. As shown in this example, orthogonalization in the presence of collinearity may result in an increased mean estimate (subject-specific estimates in row 3 of Fig 2 are too high) and a decreased variance estimate (variance of subject-specific estimates in row 3 is smaller than row 2), resulting in a higher test statistic and smaller p-value. Although the p-value for the stimulus regressor in the orthogonalized model is not appropriate for the “stimulus activation adjusted for feedback”, it is applicable to “stimulus, without adjusting for feedback activation”. This crucial difference in the interpretation of the results is unfortunately often overlooked when researchers use orthogonalization in fMRI analyses.

The above example of orthogonalization demonstrates how misleading the model results can be when orthogonalization is performed. The purpose of the present paper is to clarify when and why orthogonalization is appropriate and highlight the ways in which it affects the interpretation of results. Additionally, fMRI software packages handle orthogonalization in different ways. SPM (Statistical Parametric Mapping) automatically applies orthogonalization in certain situations, which may result in misinterpretation if users are unaware of this procedure. FSL (FMRIB Software Library) allows the user to specify what regressors are orthogonalized and so it is up to the user to do this properly. Proper uses of orthogonalization in fMRI data analysis will be discussed next, followed by the software dependent issues. Some strategies for avoiding collinearity in study design will be discussed. Finally, the overall impact that collinearity will have on group-level inferences will be described.

## Methods

### When orthogonalization is necessary

Orthogonalization is rarely warranted in fMRI analysis, but in a few special cases it is necessary in order to improve the interpretation of the model’s inferences. The simplest case is in a group level model where the BOLD contrast across subjects is modeled as a function of some parameter (e.g., age) and the inferences of interest are for the overall group mean and the age effect. In other words, the model is

$$\begin{array}{c} Y=\beta_{0}+\beta_{1}*\text{Age}+\epsilon , \end{array}$$ (2)

where *Y*, the response or explained variable, is a vector of BOLD activation values for each subject (to be “explained” by the model), Age, the explanatory variable, is the vector of ages over subjects, *β*_0_ and *β*_1_ are the parameters of interest and *ϵ* is the error term. The *β*_1_ parameter describes how much the BOLD activation changes with a 1-unit change in Age, in other words, for two subjects who are 1 year apart, the difference in their BOLD activations is *β*_1_. The other parameter, *β*_0_, is the value of the BOLD activation when Age is zero, which is not informative. However, if Age is orthogonalized with respect to the mean, this will change the interpretation of the intercept, *β*_0_, to be the overall mean, as Age will be giving up any information about the mean of the response variable. Specifically, this orthogonalization is performed by mean centering the Age variable, since the residual from the model regressing the mean from Age is *Age*_*DM*_ = *Age*−mean(*Age*). Now the GLM is

$$\begin{array}{c} Y=\beta_{0}^{*}+\beta_{1}^{*}*Age_{DM}+\epsilon . \end{array}$$

Where *Age*_*DM*_ stands for “Age de-meaned”. Although the Age regressor changed, this does not change the estimate of $\beta_{1}^{*}$ since if two subjects are 1 year apart in age, they are also 1 year apart in mean centered age, i.e. the slope parameter is unchanged. Instead, the estimate of $\beta_{0}^{*}$ now reflects the average BOLD activation, as $Y=\beta_{0}^{*}$ when *Age*−mean(*Age*) = 0 or when *Age* = mean(*Age*). In many cases there may not be any interest in the overall mean of *Y* and the extra step of mean centering age could be skipped. We highlight again here the fact that orthogonalization of age against the mean changes the parameter estimate for the mean, i.e. the intercept, not for the Age variable.

Orthogonalization is also necessary in first level fMRI designs where the model includes a parametric modulation, as shown in Fig 1, or when different trial durations are modeled, which might occur if the stimulus was shown for different periods of time across trials. In these cases a constant duration, unmodulated regressor is required (Red regressor in Fig 1). Often this regressor is of interest for studying the overall mean activation across trials, but even if of no interest it is necessary to include it. If the constant duration regressor is omitted, the model assumes that when the modulation value (i.e. intensity) is 0, the BOLD activation is 0. This is often a strong assumption and adding an unmodulated regressor will improve the model’s ability to fit the data. Using modulation by stimulus intensity as an example, the interpretation of the unmodulated regressor is the mean activation for an intensity of 0, but by orthogonalizing the modulated regressor with respect to the unmodulated regressor, the interpretation for the unmodulated regressor is the mean activation across trials. Again, the orthogonalized regressor’s inferences remain exactly the same, but the p-values and interpretation of the unmodulated regressor will change.

**Fig 3:**
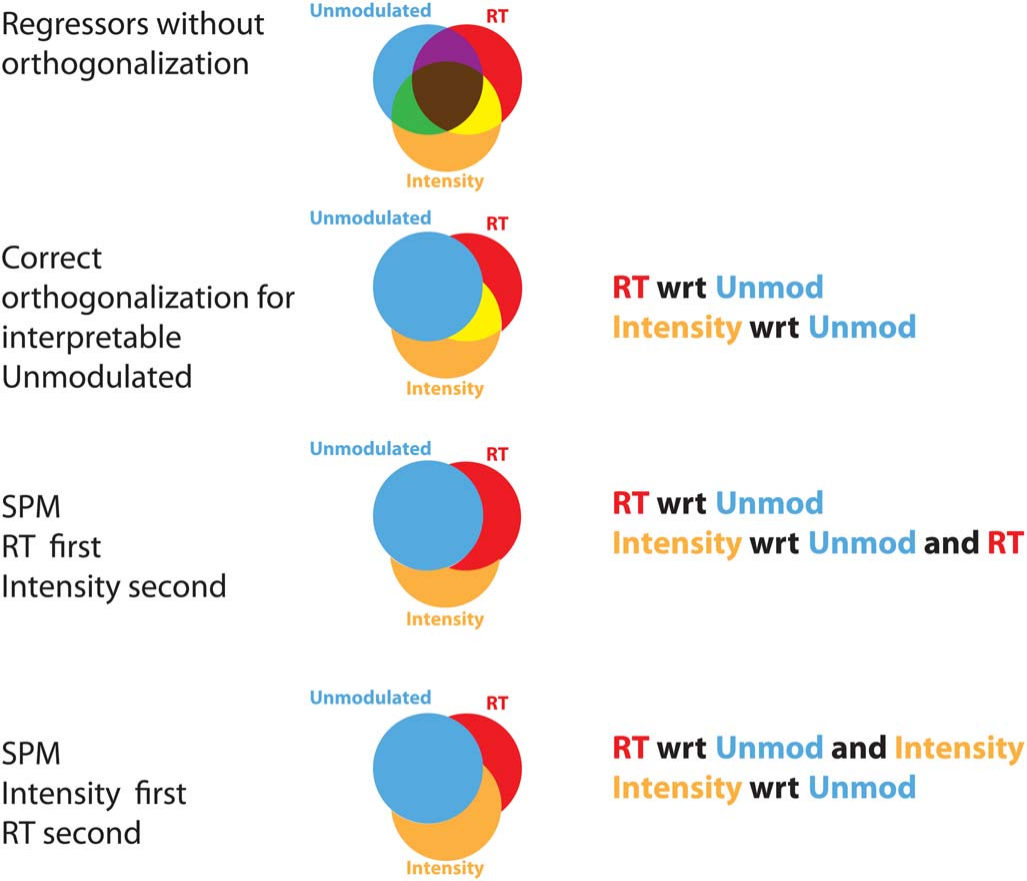
Distribution of descriptive variability with 2 sets of parametric modulators, RT and stimulus intensity. The top row shows how descriptive variability is shared when there is no orthogonalization. In this case the unmodulated regressor’s interpretation is average BOLD when RT and intensity are 0. The second row shows the orthogonalization necessary to change the interpretation of the unmodulated regressor to the average activation. Note the shared variability between RT and intensity. The last two rows show how the variability is distributed when using the automated orthogonalization in SPM when RT is entered before intensity and vice versa. The modulation that is entered first is not adjusted for the second, which can lead to misleading results. SPM sequentially orthogonalizes a modulated regressor with the previous regressors, therefore two models may have to be run to obtain the specific variance of two modulations.

Fig 3 extends this example to when there are 2 modulation values: RT and stimulus intensity. The top row illustrates how the descriptive variability is split across the 3 explanatory variables where there are 3 unique portions and 4 shared portions. The interpretations for the RT and intensity regressors are sensible, but the unmodulated regressor’s interpretation is the mean activation when both RT and stimulus intensity are 0, which is not of interest. In this case both the RT and intensity modulated regressors would be orthogonalized with respect to the unmodulated regressor to assign all variability shared with the unmodulated regressor to it (second row, Fig 3). Now each parameter has the proper interpretation. The unmodulated regressor is the overall mean BOLD activation across trials, the RT effect is adjusted for intensity and the intensity effect is adjusted for RT.

### Orthogonalization in fMRI software

#### SPM

The SPM software package automatically performs orthogonalization for parametrically modulated regressors in versions 8 and older. However, due to how orthogonalization is performed when more than one set of parametric modulation values is used for a set of trials, it is possible to misinterpret the resulting inferences. In the case of a single parametrically modulated value per trial, for example RT, SPM first creates the unmodulated regressor and next creates a regressor where each trial is modulated by the reaction time. In this case the modulated regressor is automatically orthogonalized with respect to the unmodulated regressor. As discussed above, this is an appropriate use of orthogonalization for improving the interpretation of the unmodulated regressor.

As described in the previous section (second row, Fig 3) both RT and stimulus intensity should be orthogonalized with respect to the unmodulated regressor to improve the interpretation of the unmodulated regressor. Instead, SPM (up to version 8) orthogonalizes each regressor with respect to the ones preceding it; thus, the results will vary depending upon the order in which the regressors are specified in the model. If the RT modulations are entered prior to the intensity modulations, RT is orthogonalized with respect to the unmodulated regressor and stimulus intensity is orthogonalized with respect to the unmodulated regressor *and* RT. As shown in the third row of Fig 3, RT is no longer adjusted for stimulus intensity. Since RT was given the shared descriptive variability (the yellow portion) it is likely that the p-value for RT in the 3rd row will be smaller than that of the correct model in the second row. This paired with the incorrect interpretation that the inference applies to RT adjusted for stimulus intensity can yield misleading results in publications. Likewise, if stimulus intensity is entered first, followed by RT, the intensity effect is no longer adjusted for RT (last row, Fig 3).

The solution to this problem is to circumvent the automated procedure. One possibility would be to run 2 models, one where RT was the second modulation entered and one where stimulus intensity amount was the second modulation, in order to obtain the appropriate statistics for the adjusted RT and stimulus intensity effects, respectively. Another solution is to create modulated regressors outside of the GUI and enter them as user-specific regressors in the SPM analysis. If using SPM12, there is now an option to orthogonalize within the graphical user interface.

One other instance in which orthogonalization is automatic in SPM is when derivatives of regressors are added to a model. This is typically done in an effort to adjust for minor misspecifications in the stimulus timing. In SPM the regressor corresponding to the first derivative of the regressor is orthogonalized with respect to the original regressor. As illustrated in [2], this can have a beneficial impact on the overall model fit and the estimate of the original regressor, which is typically the regressor of interest.

#### FSL

The FSL package does not perform automatic orthogonalization, but it does allow for user-specified orthogonalization. It is up to the user to properly orthogonalize regressors when necessary. To do so, a button marked “Orthogonalise” would be clicked and then the regressors to orthogonalize with respect to would be checked. Note, the regressor for which “Orthogonalise” is selected is giving up its shared variability to the other checked regressor(s). The parameter estimates and inferences for the orthogonalized regressor will remain exactly the same while the regressors corresponding to the boxes that are checked in the GUI will change if they are correlated. Care must be taken in interpreting any regressor to which another has been orthogonalized, as it is no longer adjusted. For example, if the motion parameters are orthogonalized with respect to task, the task effect is no longer adjusted for motion, which can lead to inflated effect sizes due to artifact in the data if the convolved task is correlated with motion.

Of note, it is common practice to mean center the modulation values in the behavioral onset file that is fed into an FSL analysis and this will remove collinearity between the parametrically modulated regressor and the constant duration regressor. In this case, orthogonalization is not necessary. It is most commonly used in first level models where the duration of a trial varies across trials.

Similar to SPM, FSL also allows for the modeling of temporal derivatives of regressors to account for minor timing misspecifications. FSL uses a slightly different orthgonalization strategy where the derivative is orthogonalized with respect to both the original regressor and the overall mean. As shown in [2], this approach may still be beneficial in improving the estimate for the regressor of interest, the original regressor, but not as beneficial as only orthogonalizing with respect to the original regressor, as done in SPM.

#### AFNI

The AFNI package does not appear to have specific tools for orthogonalization. It does include a tool (xmat_tool.py) that can display the correlation matrix for the model regressors.

### Avoiding collinearity

Collinearity in a study design can be avoided if, during the study planning phase, efficiency calculations are be used to identify the most efficient design and reduce any collinearity issues. Efficiency calculations can be used to compute a numeric value associated with the unique descriptive variability associated with each parameter estimate, as shown in the Venn diagrams above. Specifically, the efficiency for a specific contrast of parameters, say if the alternative hypothesis is whether *cβ* > 0, for some contrast row-vector, *c*, the efficiency is given by

$$\begin{array}{c} \text{eff}\left(c\beta \right)=1/c{\left(X^{\prime }X\right)}^{-1}c^{\prime } \end{array}$$ (3)

**Fig 4:**
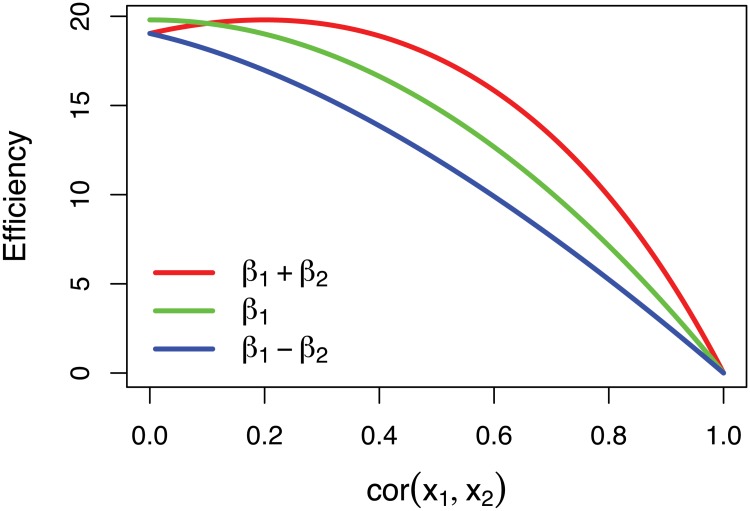
Efficiency as a function of correlation between two regressors for a single parameter (green), the sum of parameters (red) and their difference (blue). As collinearity increases, the efficiencies of all three of these contrasts of parameter estimates diminishes.

[3]. For simplicity, assume there are only two regressors in the model, *x*_1_ and *x*_2_ with corresponding regression parameters *β*_1_ and *β*_2_ where the interest is maximizing the efficiency for *β*_1_. Assuming the regressors are mean centered, it can be shown that the efficiency for *β*_1_ is

$$\begin{array}{c} \text{eff}\left(\beta_{1}\right)=\left(N-1\right)\text{Var}(x_{1})(1-cor^{2}(x_{1},x_{2})), \end{array}$$ (4)

where *N* is the number of observations. This equation illustrates that if the variance of *x*_1_ is fixed, when the correlation between *x*_1_ and *x*_2_ increases, the efficiency decreases. This relationship is illustrated by the green line in Fig 4 of efficiency as a function of correlation Since a typical efficiency calculation fixes the number of trials, but simply randomizes trial order until the most efficient design is found, the variance for *x*_1_ and *x*_2_ will be fixed across all candidate designs, implying the most efficient design that is identified will be the design with the lowest correlation between *x*_1_ and *x*_2_. Also of interest may be the sum or difference of parameter and the red and blue lines in Fig 4 illustrate that these contrasts of parameter estimates are also negatively impacted by collinearity between the explanatory variables.

To reduce collinearity and increase efficiency the ISI can be increased. Varying the ISI (e.g., randomly drawing from a distribution such as the truncated exponential) will also help reduce collinearity between adjacent trials, as in the stimulus/feedback cue model discussed earlier. There will often be a slight tradeoff in obtaining a design with low collinearity while staying within a reasonable range of ISIs for psychological criteria to be met. For example, if a stimulus and feedback are very close, the separate BOLD signals cannot be determined, but if they are separated by a large ISI, this may impede learning. Given this information, it is also helpful if any behavioral studies run prior to the imaging portion of the study also take these stimulus timing issues into consideration in order to keep the initial behavioral results consistent with what will occur in the scanner. Even though these collinearity issues are not a problem in a strictly behavioral analysis, if behavior effects are diminished after optimizing a behavioral study for the scanner, the fMRI effects may also diminish.

Although efficiency can be used to find the best design within a subset of possible designs, the measure alone does not indicate the degree of collinearity. If the parameters set for the model search only include highly collinear designs, the search will find a most efficient design, but it is not guaranteed to be free of collinearity. In addition to traditional efficiency calculations, the variance inflation factor (VIF) can be used to study collinearity directly. The VIF for a regressor *X*_*i*_ is defined by ${\text{VIF}}_{i}=1/(1-R_{i}^{2})$, where $R_{i}^{2}$ is the *R* ^2^ from the model where the regressor of interest is modeled as a function of all other regressors (*X*_*i*_ = *β*_1_ *X*_1_+…+*β*_*i*−1_ *X*_*i*−1_+*β*_*i*+1_ *X*_*i*+1_+…+*β*_*N*_ *X*_*N*_+*ϵ*) (wikipedia.org/wiki/Variance_inflation_factor). If the *R* ^2^ value is high, the VIF will also be high, indicating the regressor of interest is a linear combination of the other regressors and a collinearity problem is present. Typically a cutoff of 5 or 10 is used as a threshold for problematic collinearity. This rule of thumb however is criticized by [4] who note that the VIF should be considered in the context of group sizes. In practice, it would be useful to record efficiency as well as VIF for the candidate designs in order to maximize efficiency while guaranteeing a low level of collinearity. In addition, if the study has already been carried out, the VIF can help assess the degree of the collinearity.

### Impact of collinearity on inferences

Collinearity can be problematic when interpreting model results, and a common misunderstanding in fMRI analysis is that collinearity fully ruins any ability to interpret the results. In all cases of collinearity inferences are valid, meaning the type I error rate is controlled. The primary worry is that the highly variable parameter estimates may be much larger or smaller than the true magnitude, but when collinearity causes this to occur in the model the estimated variance is also large. Thus, although the parameter estimates are highly variable, they are unbiased and Type 1 error rate is preserved, so inferences remain valid in the presence of collinearity [5]. This means if multiple estimates from independent studies (or subjects) are averaged, the mean will converge on the true value. Most fMRI analysis software packages use a multistage approach to analyze data, otherwise known as the summary statistics approach to the mixed model [6, 7]. The first stage of analysis involves analyzing each run of data independently and, assuming there is a single run per subject, the second stage combines first level estimates in a group model. The impact of collinearity depends on what stage of modeling the collinearity occurred (single subject or in group model). If the collinearity occurs in the first level, say if two explanatory variables for two trial types are correlated and the interest is in the effect of the first trial type, the individual subject parameter estimates will be highly variable, but when averaged at the group level the estimates that are too large tend to balance out with those that are too small to arrive at an estimate that is closer to the true mean estimate. On the other hand, collinearity between age and gender would occur in the group model and in this case the parameter estimate with, say, age may be much larger or smaller than the true effect such that if the magnitude of the effect is of great interest, care should be taken in interpreting it. The confidence interval associated with the estimate will reflect the wide range of parameter values that are consistent with the data, so if a region of interest analysis is performed this should be included as well. Note that this would also be the case in analyses, such as VBM (voxel based morphometry), where there is only one stage in the analysis analyzing relationships between brain measures and sets of covariates that tend to be highly correlated. The inferences are valid, but in the presence of collinearity, the parameter estimates will be highly variable.

## Conclusions and Discussion

Even the best planned studies can result in models with collinear regressors. Although the use of orthogonalization may be a tempting way to remove collinearity from the model, it does not change the overall fit of the model and in most cases it confuses the interpretation of the resulting inferences. When applying orthogonalization, if automated by data analysis software or manually done by a user, it is important to understand the impact this has on the inferences associated with that model when reporting results. Orthogonalization should only be used to improve the interpretation of regressors, such as in the case of mean centering a continuous covariate so that the mean regressor is interpreted as the overall mean.
